# VOICES FROM THE FIELD: EXPLORING THE VALUE OF GERONTOLOGISTS IN INTERPROFESSIONAL GERIATRIC CARE TEAMS

**DOI:** 10.1093/geroni/igae098.4097

**Published:** 2024-12-31

**Authors:** Alfred Boakye, Diane Martin

**Affiliations:** University of Maryland Baltimore, Baltimore, Maryland, United States; University of Maryland Baltimore, Baltimore, Maryland, United States

## Abstract

Interprofessional healthcare teams effectively deliver safe and high-quality care to patients with complex healthcare needs. Through collaborative team-based efforts, doctors, nurses, social workers, pharmacists, and other professionals address multiple aspects of health, including physical, mental, and social well-being. Team-based care is now recognized as a national priority to optimize the health and social well-being of older adults and their families; however, geriatric care teams often do not include professionals with expertise in aging, such as gerontologists. The aim of this study was to explore what gerontologists can contribute to a geriatric interprofessional care team. Sixteen (16) gerontology students, faculty, and healthcare professionals completed semi-structured surveys, and the results were analyzed using a modified grounded theory. Respondents highlighted that the unique interdisciplinary perspective on the aging process held by gerontologists can promote person-centered care and bridge the gap between older adults and community resources. These findings illustrate the valuable role that gerontologists can have as members of interprofessional geriatric healthcare teams. Additional research is needed to evaluate whether integrating gerontologists can lead to enhanced care, improved care coordination, and advance evidence-based practices, research, and policy.

